# The Bleeding Bowel: A Rare Case of Neurofibromatosis Type 1-associated Gastrointestinal Stromal Tumor in a Young Male

**DOI:** 10.7759/cureus.4868

**Published:** 2019-06-10

**Authors:** Ricci Kalayanamitra, Zainab Shahid, Neal Shah, Ravi Patel, Rohit Jain

**Affiliations:** 1 Emergency Medicine, Penn State Health Milton S. Hershey Medical Center, Hershey, USA; 2 Internal Medicine, Lake Erie College of Osteopathic Medicine, Erie, USA; 3 Internal Medicine, Penn State Health Milton S. Hershey Medical Center, Hershey, USA

**Keywords:** neurofibromatosis type 1, gastrointestinal stromal tumor, gist, nf1, nf1-associated gist, gi bleed, gastrointestinal bleed, melena, anemia, young

## Abstract

Individuals with neurofibromatosis type 1 are much more likely to develop gastrointestinal stromal tumors than those without this condition. The median age for patients with neurofibromatosis type 1-associated gastrointestinal stromal tumors is approximately 65 years. We present a case of a young male with a history of neurofibromatosis type 1 who presented with symptomatic anemia and melena and was ultimately found to have a gastrointestinal stromal tumor.

## Introduction

Neurofibromatosis type 1 (NF1), also known as von Recklinghausen's disease, is the most common form of the three major clinically and genetically distinct classifications of neurofibromatosis (type 1, type 2, and schwannnomatosis) [1]. It is an autosomal dominant condition that is caused by loss-of-function mutations in the tumor-suppressor gene NF1 and is characterized by cutaneous pigmentations and growth of tumors along nerves in the skin, brain, and other parts of the body [2]. According to the practice guideline for treating adults with NF1 by Medical Genetics and Genomics (ACMG) published in 2018, up to 1 in every 1,900 people is affected by NF1 worldwide. NF1 has been shown to decrease the average life expectancy of those affected by 8 to 15 years, most commonly due to malignancies and cardiovascular complications [3].

NF1 has a predilection for cancer development with a five-fold increase in overall cancer risk, which is the highest cancer incidence yet reported. It is closely associated with malignant peripheral nerve sheath tumors, breast cancers, and pheochromocytomas [4]. It has also been found to be associated with malignant fibrous histiocytomas, thyroid carcinomas and gastrointestinal stromal tumors (GISTs). The estimated cumulative cancer risk in patients with NF1 was 25.1% by age 30 years and 38.8% by age 50 years, whereas the respective percentages in the general population of the same region were 0.8% and 3.9%. The estimated lifetime cancer risk in patients with NF1 is 59.6%, while it is approximately 38.5% in the general population [4-5].

Patients with NF1 are 34 times more likely to develop GISTs than those without NF1 [6]. Although rare, GISTs are the most common mesenchymal tumors within the gastrointestinal tract [7]. A systematic review of 29 population-based studies involving 13,550 patients from 19 countries found that the reported incidence of GISTs overall was 10-15 people per million per year [8]. In contrast, the United States Cancer Statistics database from 2001 to 2015 revealed that the annual incidence of GISTs in the United States was seven cases per million people [9]. The primary locations of GISTs were 55.6% gastric, 31.8% small bowel, 6.0% colorectal, and 6.6% others. The ages of patients with GISTs ranged from 10 to 100 years, with the median age being roughly 65 years [8].

## Case presentation

A 21-year-old male with a past medical history of NF1 presented with shortness of breath, lethargy, and melena. He had been admitted to an outside hospital (OSH) two weeks prior with similar symptoms and was found to be anemic due to a gastrointestinal bleed. Colonoscopy and esophagogastroduodenoscopy (EGD) did not reveal any pathology, so the patient was discharged and scheduled for an outpatient capsule endoscopy. This was not performed due to his abrupt re-hospitalization to the OSH due to tachycardia and anemia with hemoglobin of 6 g/dL. Laboratory workup was non-contributory, so the patient was transfused with three units of packed red blood cells and was transferred to our institution [10].

**Figure 1:**
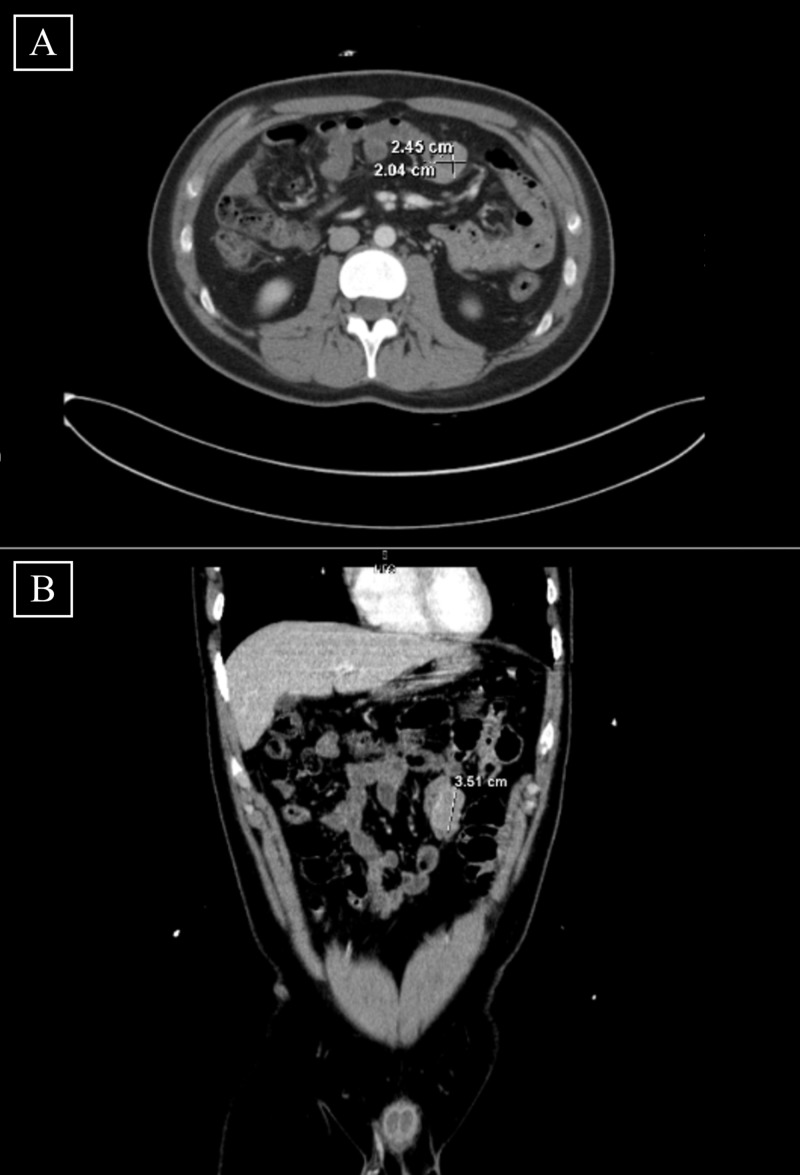
[A] Transverse view of CT scan revealing a 2.45 cm x 2.04 cm well-circumscribed ovoid mass in the jejunum. [B] Coronal view of CT scan revealing a 3.51 cm well-circumscribed ovoid mass in the jejunum. CT: Computed tomography

The patient reported continued lethargy and denied abnormal bowel movements between his two earlier hospitalizations. His abdominal and rectal examinations were unremarkable, and skin examination revealed café-au-lait spots on the right arm. Hemoglobin was 8.5 mg/dL, total bilirubin was 2.3 mg/dL, and direct bilirubin was 0.3 mg/dL. Vital signs were within normal limits. An abdominal computed tomography (CT) scan revealed a 2.45 x 2.04 x 3.51 cm well-circumscribed ovoid mass at the jejunum adjacent to a loop of small bowel in the left upper quadrant (Figures 1A, 1B). This mass was subsequently resected, biopsied, and confirmed as a low-grade GIST [10]. The surgical oncology team determined that he did not require any adjuvant chemotherapy or immunotherapy because his tumor was classified as low risk. The patient was ultimately discharged with pain control medications and was followed by his OSH care providers for any recurring mass. At one-year follow-up, he remained completely asymptomatic and free from cancer.

## Discussion

The differential diagnoses of patients who present with anemia are broad. In the setting of anemia with melena, CT scans, EGDs, and colonoscopies are often ordered. If these examinations are non-revealing, patients are subjected to repeat studies or more specialized studies, such as capsule endoscopies and balloon enteroscopies. This adds to the physical and financial burdens of patients and may still fail to identify the underlying etiology of their symptoms. After admitting the patient in this case to our institution, we had initially planned for a push enteroscopy and a capsule study but were able to avoid this course by taking a thorough history of his NF1, researching its link to GISTs, and correlating this with the suspicious findings on imaging [10].

Without understanding this connection between NF1 and GISTs, achieving a prompt diagnosis would have been difficult given the patient’s limited number of symptoms. Although our patient endorsed fatigue and had melena, he lacked abdominal pain, nausea, vomiting, bowel obstruction, a palpable abdominal mass, early satiety, dysphagia, and odynophagia, all of which have been reported as the most common signs and symptoms of GISTs [11]. His lack of symptoms is not unique, as a patient series conducted on 15 patients with NF1-associated GISTs found that GISTs were incidentally detected among seven patients while the other eight had symptomatic tumors [12]. This further accentuated the importance of linking NF1 and GISTs because it demonstrated that patients with NF1-associated GISTs could be asymptomatic.

Identifying this tumor early allows for prompt intervention and potential cure or halt of disease progression because GISTs are treatable tumors. Approximately 60% of GISTs are cured with surgery alone, especially in cases that involve low-to-moderate risk GISTs. With higher risk GISTs, a randomized phase III multi-center clinical trial demonstrated that patients who underwent surgery had significantly better recurrence-free survival and overall survival rates with three years of adjuvant imatinib therapy when compared to those who had only one year of adjuvant imatinib therapy [13]. Despite its rarity, providers should consider GISTs in patients with a history of NF1 who present with gastrointestinal symptoms, especially because there are effective treatment options for it.

This clinical suspicion should not be limited to older patients, despite the estimated median age for NF1-associated GISTs being 65 years, as the patient in this report was significantly younger [8]. A population-based prospective cohort study conducted on 1,404 NF1 patients from 1987 to 2012 reported 217 deaths throughout the study, with 107 (49%) of them being due to cancer. The mortality rate was reported to be highest in young adulthood, between ages 15 and 30 years [4]. In addition, a previous case report highlighted the ability of NF1-associated GISTs to recur throughout a patient’s life [14]. This, along with the mortality rate being higher in younger adults with NF1-associated GISTs, makes detecting the tumor in younger patients even more crucial. After having his GIST detected and removed at a young age, our patient can now be routinely monitored for any recurring mass throughout his life.

## Conclusions

Providers should have a high index of suspicion for GISTs when taking care of patients with NF1 who present with gastrointestinal symptoms, regardless of age and/or a limited number of symptoms. Early detection of GISTs can minimize the financial and physical burdens associated with unnecessary diagnostic tests. Furthermore, since GISTs are treatable tumors, a prompt diagnosis can lead to effective treatment and prevention of disease progression.
